# Epigenetic clocks can differentiate between exogenous and endogenous skin ageing

**DOI:** 10.1186/s13148-026-02230-w

**Published:** 2026-09-11

**Authors:** Christopher J. Shore, Roy B. Simons, Laura Ramos del Caño, Ziyi Xiong, Luba Milena Pardo, Christina M. W. Eecen, Tamar Nijsten, Kerrin S. Small, Athina Vidaki, Manfred Kayser, Jordana T. Bell, Y. Jeremy Shen, David A. Gunn

**Affiliations:** 1 Department of Twin Research, Ageing and Genomic Epidemiology, King’s College London, London, UK; 2 Department of Pathology and Clinical Bioinformatics, Erasmus MC University Medical Center Rotterdam, Rotterdam, The Netherlands; 3 Unilever R&D at King’s College London, London, UK; 4 Department of Dermatology, Erasmus MC University Medical Center Rotterdam, Rotterdam, The Netherlands; 5 Department of Genetic Identification, Erasmus MC University Medical Center Rotterdam, Rotterdam, The Netherlands; 6 Unilever R&D, Trumbull, Connecticut USA; 7 King’s College London, London, UK; 8 Department of Biotechnology, Delft University of Technology, Delft, The Netherlands; 9 Department of Clinical Genetics, Maastricht University Medical Center, Maastricht, The Netherlands

## Abstract

**Context:**

Whereas factors originating internally to the body, such as blood nutrients and hormones, influence endogenous skin ageing, exogenous skin ageing results from the penetration of harmful factors through the skin’s outer stratum corneum layer.

**Aim:**

To test whether an exogenous skin ageing epigenetic clock is more strongly associated with aspects of sun-exposure than two endogenous epigenetic clocks.

**Methods:**

Exogenous and endogenous skin clocks were trained in more than 400 skin samples. These two clocks and the Horvath ‘Skin and Blood’ epigenetic clock were tested for association with chronological age and, whilst controlling for chronological age (age acceleration), sun-exposure parameters and skin gene expression.

**Results:**

Exogenous Skin clock ages were significantly (*p*<0.001) older in both epidermal (n=24) and cultured fibroblast (n=8) sun-exposed arm samples than in donor matched sun-protected samples. Exogenous Skin clock age acceleration was significantly correlated (Rho = 0.18, *p* = 0.004) with sun-seeking behaviour and mRNA levels (202 genes had q < 0.05) in whole skin abdomen samples (n=364); the latter was consistent with sun-exposed versus sun-protected older arm mRNA differences (n=12 donors). In 44 facial skin swabs, exogenous age acceleration was also significantly correlated with facial photodamage grading (rho = 0.33, *p* = 0.028). Although the Endogenous and Skin and Blood clocks were more strongly correlated with chronological age, their age acceleration estimates were not significantly associated with sun-exposure parameters or mRNA levels in skin.

**Conclusion:**

Skin epigenetic clocks can differentiate exogenous from endogenous skin ageing facilitating further study of the mechanisms underlying these two skin ageing phenomena.

**Electronic supplementary material:**

The online version of this article (https://doi.org/10.1186/s13148-026-02230-w) contains supplementary material, which is available to authorized users.

## Background

Skin ageing is associated with a wide variety of phenotypic changes which contribute to, in addition to cosmetic changes, increased risk of skin diseases in later life. As such, skin ageing represents a substantial burden on both individuals and healthcare systems (Ibrahim et al. 2023). In an increasingly ageing population, biomarkers for quantifying the rate of skin ageing could play an important role in numerous clinical applications and for commercial purposes.

In skin, the organ which is most exposed to the external environment, ageing can be attributed to endogenous and exogenous derived factors. Endogenous skin ageing can be defined as ageing attributable to factors originating internally to the body (e.g. metabolic stress, blood nutrient supply), while exogenous skin ageing refers to ageing driven by factors penetrating the skin through the stratum corneum, the outermost layer of the epidermis. According to this distinction, endogenous skin ageing is expected to influence ageing across all skin body sites whereas exogenous skin ageing should predominantly impact ageing in environmentally exposed body sites such as the face. Importantly, the phenotypes in exposed and protected aged skin differ somewhat, with exposed skin having an increased risk of skin cancers such as melanoma (D’Arino et al. 2023; Trojahn et al. 2015), particularly in lighter skinned individuals with relatively high sun-exposure. Previous studies have identified phenotypic and molecular changes associated with endogenous and exogenous skin ageing by comparing skin in sun-protected sites to sun-exposed sites (Bormann et al. 2016; Vandiver et al. 2015; Yan et al. 2024), although this is complicated by potential interactions between the two processes in exposed sites. Hence, distinguishing endogenous and exogenous ageing in sun-exposed skin remains a challenge in skin ageing research.

DNA methylation (DNAm) is the most studied epigenetic modification, involving the addition of a methyl group to the 5’ carbon of the cytosine residue in a cytosine-phosphate-guanine (CpG) dinucleotide. One of the most notable functions of DNAm is the regulation of gene expression, with DNAm in gene promoters typically being associated with downregulation of gene expression, albeit often in context-dependant settings (Sergeeva et al. 2023)*.*

Previous studies have shown that DNAm patterns are highly associated with chronological and biological ageing (Horvath 2013; Hannum et al. 2013; Levine et al. 2018; Lu et al. 2019), and skin-specific studies have identified DNAm changes associated predominately with either endogenous or exogenous skin ageing (Vandiver et al. 2015; Yan et al. 2024; Jeremian et al. 2024). To enable the differentiation between endogenous and exogenous ageing, skin ageing changes in protected sites are compared with changes in exposed sites, thus revealing changes occurring either entirely or to a significantly greater extent because of environmental exposure.

DNAm is strongly associated with chronological age and has been used to develop biological age prediction models, termed epigenetic clocks. An array of epigenetic clocks specific to blood and multiple tissues have been widely studied across multiple phenotypes, showing strong associations with many age-related exposures and diseases (Horvath 2013; Hannum et al. 2013; Levine et al. 2018; Lu et al. 2019). Some clocks have been developed specifically for skin, such as the Horvath “Skin and Blood” epigenetic clock (Horvath et al. 2018) and the epigenetic clock generated by Boroni et al., (2020). However, both of these skin epigenetic clocks were trained to predict chronological age of tissue donors. Specifically, the Skin and Blood clock uses DNAm profiles of blood, buccal, nasal epithelium, sun-exposed epidermis, and cultured fibroblasts from sun-protected and sun-exposed skin sites (Horvath et al. 2018). Similarly, Boroni et al., (2020) use DNAm profiles from a mixture of sun-exposed and sun-protected whole skin, dermis, and epidermis. As such, both current skin epigenetic clocks are likely heavily biased towards endogenous skin ageing.

At least one skin epigenetic clock has been trained to predict visual facial age: the VisAgeX clock (Bienkowska et al. 2024). This clock this may capture more aspects of exogenous ageing compared to aforementioned epigenetic clocks. However, as visual facial age is driven by both endogenous and exogenous factors (Trojahn et al. 2015), the VisAgeX clock is likely capturing a mixture of exogenous and endogenous skin ageing factors. Therefore, it is unlikely to distinguish between these two types of skin ageing, particularly in non-facial skin sites.

Furthermore, endogenous and exogenous skin ageing are subject to different clinical and cosmetic interventions. As such, epigenetic clocks that can differentiate between endogenous and exogenous skin ageing would be of great value in determining which treatments work best for exogenous and endogenous skin ageing and could enable personalised clinical and cosmetic advice and interventions. These epigenetic clocks would also have valuable research applications, such as providing insight into the hereto relatively unknown molecular mechanisms of many skin ageing interventions, including retinoic acid to treat severe photoageing (Mambwe et al. 2025) and vitamin B3 used to reduce risk of skin cancers (Ong and Goh 2024). Finally, these epigenetic clocks could be used in the development of new clinical and cosmetic therapeutics, specifically in the context of testing the efficacy of a therapeutic in targeting endogenous and/or exogenous skin ageing.

Here, we present the Exogenous Skin epigenetic clock, which is tailored towards predicting exogenous ageing in skin, and the Endogenous Skin epigenetic clock, which is tailored towards predicting endogenous ageing in skin. We validate these epigenetic clocks in several independent datasets, including whole-biopsy (combined epidermis and dermis) skin, epidermal skin, and dermal fibroblast cultures. We then describe an adjustment of the Exogenous Skin clock which improves its accuracy in assessing photoageing in dermal fibroblasts. Finally, we examine the biological insights which might be gained from these clocks by analysing skin gene expression data.

## Materials and methods

See Tables 1, 2, and 3 for the use of each dataset and a description of each.

**Table 1:** Training datasets for the exogenous skin clock. The fibroblast data was only used for the re-training of the exogenous model

Dataset	Reference ID	Samples/Donors	Tissue/Cell	Body Site	Platform	Mean age (range)
Bormann dataset	E-MTAB-4385 (Bormann et al. 2016)	59/59	Epidermis	Sun-exposed outer-forearm	Infinium 450k array	49.3 (21–76)
Furtak young old dataset (sun-exposed data only)	E-MTAB-14619 (Furtak *et al.*, 2026)	48/48	Whole skin	Sun-exposed outer-forearm	Infinium 450k array	40.3 (21–65)
Fibroblast exposed dataset (‘re-trained’ clock only)	E-MTAB-15155	7/7	Dermal Fibroblasts	Sun-exposed outer-forearm	Infinium 450k array	50.9 (21–82)

**Table 2:** Test datasets for the Exogenous, Endogenous and Skin and Blood clocks. All three clocks were tested on all datasets. The MuTHER dataset was also used as training for the endogenous clock (see Table 3) and, thus, ages will not be representative of a true test dataset for this clock

Dataset	Reference ID	Samples/Donors	Tissue/Cell	Body Site	Platform	Mean age (range)
Vandiver dataset	GSE52980 (Vandiver et al. 2015)	78/20	Dermis and Epidermis	Sun-exposed outer-forearm (N = 29) or lateral epicanthus (N = 10) and Sun-protected inner forearm (N = 39)	Infinium 450k array	50.7 (20 – > 90)
MuTHER dataset:	EGAS00001007816 (Shore et al. 2024; Nica et al. 2011)	414/414	Whole skin	Abdomen, adjacent to umbilicum	Infinium 450k array	58.9 (38.7–84.6)
Rotterdam Study dataset	N/A	44/44	Skin swabs	Naso-labial fold	Targeted DNAm Ion Ampliseq	54.2 (45–69)
Fibroblast young old dataset	E-MTAB-15156	42/7	Dermal Fibroblasts	Sun-exposed outer-arm (N = 21) and sun-protected inner-arm (N = 21)	Infinium 450k array	51.2 (21–78)

**Table 3:** Training datasets for the endogenous skin clock. The same testing datasets from Table 2 were also used for testing the endogenous skin clock

Dataset	Reference ID	Samples/Donors	Tissue/Cell	Body Site	Platform	Mean age (range)
Bormann dataset	E-MTAB-4385 (Bormann et al. 2016)	59/59	Epidermis	Sun-exposed outer-forearm	Infinium 450k array	49.3 (21.9–76.0)
Furtak young old dataset (sun-protected only)	E-MTAB-14619 (Furtak et al., 2026)	48/48	Whole skin	Sun-protected upper inner arm	Infinium 450k array	40.3 (21–65)
MuTHER dataset (subset of 48; Table S1)	GSE90124 (Roos et al. 2017; Nica et al. 2011)	48/48	Whole skin	Abdomen, adjacent to umbilicum	Infinium 450k array	60.3 (39–78)
Shyne dataset	Currently under embargo (Shyne et al. 2024)	24/24	Epidermis	Upper buttock	Infinium EPIC array	47 (19–81)

### Whole biopsy skin datasets

#### Young and old, lower outer and upper inner arm whole-biopsy skin DNAm profiles - Furtak young old dataset

This dataset consisted of 96 Illumina Infinium HumanMethylation450 BeadChip (Illumina 450k) array-based epigenetic profiles generated from 48 whole-skin biopsies taken from sun-exposed outer forearm, and 48 whole-skin biopsies taken from sun-protected inner arm. These were collected from both young and old individuals in a cohort of Chinese and European female donors aged between 21 and 65 years. Data is available in Array express (ID E-MTAB-14619); see Furtak et al. 2026 for further details.

#### Abdomen whole skin RNA-seq and DNAm profiles – MuTHER dataset

Two subsets of whole-skin DNAm profiles, profiled from TwinsUK participants as part of the Multiple Tissue Human Expression Resource (MuTHER) study (Nica et al. 2011; Verdi et al. 2019), and an overlapping subset of TwinsUK whole-skin RNAseq profiles (Buil et al. 2015), were used in this study. The first subset, used for training the Endogenous Skin clock, consists of 48 whole-skin DNAm profiles, a subset of 322 profiles described previously by Roos et al., (2017), which is available from the Gene Expression Omnibus (GEO, accession number GSE90124).

The second subset consists of 414 whole-skin DNAm profiles (available on the European Genome-Phenome Archive (EGA); accession: EGAS00001007816), and an overlapping set of 364 whole-skin RNA-seq profiles (available on EGA; accession: EGAS00001000805), previously described by Shore et al., (2024), and Buil et al., (2015), respectively.

These datasets have been previously described in detail (Nica et al. 2011; Buil et al. 2015; Roos et al. 2017; Shore et al. 2024). Briefly, whole-skin punch biopsies were taken from a relatively sun-protected region adjacent and inferior to the umbilicum from donors in the TwinsUK cohort (Verdi et al. 2019). These biopsies were then separated into a skin and adipose tissue portion, and then the skin tissue was again divided in two. One of these two halves of the whole-skin biopsy was used for DNAm profile generation using the Illumina Infinium HumanMethylation450 BeadChip Platform, while the other half was used for RNA-seq profiles generated using the Illumina HiSeq 2000 platform.

Of the 414 individuals in the larger subset of whole-skin DNAm data (EGAS00001007816), 242 had existing sun-seeking behaviour data, measured by the number of weeks spent abroad in hot climates (Sanna et al. 2021). Specifically, these individuals previously completed a questionnaire which asked: “How many weeks have you spent in hot countries?” cumulatively throughout their lifetime. Although this data was collected in the late 1990s, ~10 years prior to the collection of whole-skin biopsies in the late 2000s, we may assume that patterns of sun-seeking across the lifetime of an individual are relatively stable, and that this variable captures to an extent the degree to which an individual exposes their abdominal skin to sunlight. This variable was log1p-transformed prior to analysis.

#### Whole-biopsy skin lower outer and upper inner arm RNA-seq profiles – RNA-seq dataset

This dataset consists of 24 whole-skin RNA-seq profiles from twelve older (age range: 55–70) female donors with Fitzpatrick skin types I–III. Biopsies were collected by proDERM GmbH in Germany. For each donor, 3mm full-thickness skin biopsies were taken from the lower outer and upper inner arm. All donors gave written informed consent, and ethical approval was given by the institutional Review Board of proDERM GmbH. Samples were embedded in FFPE blocks and sectioned into 10um paraffin slices and stored at − 80 °C before total RNA extracted using the FormaPure RNA XL Extraction Kit (Beckman Coulter, PN: C36001) following manufacturer’s instructions. RNA quality was assessed on a Fragment Analyzer System using the DNF-471 Agilent RNA Kit. Sequencing libraries were prepared using the QuantSeq 3’ mRNA-Seq Library Prep Kit FWD with UDI and sequencing was performed on an Illumina NextSeq 2000 platform. The RNA data is available in ArrayExpress under E-MTAB-15291.

### Epidermal and dermal datasets

#### Lower outer arm epidermal skin DNAm profiles across an age range - Bormann dataset

This dataset consists of epigenetic profiles of 24 young (18–27 years) and 24 old (61–78 years) sun-exposed outer forearm skin biopsies as well as suction blister roofs from sun-exposed forearm sites taken from 60 subjects aged 20–79 years. These profiles were Illumina 450k array-based and have previously been described in detail by Bormann et al., (2016). A subset of 59 samples, ranging from 21 to 76 years of age, was downloaded from ArrayExpress (accession: E-MTAB-4385) and employed for training both the Endogenous and Exogenous Skin clocks.

#### Upper buttock sun-protected epidermal DNAm profiles – Shyne dataset

This dataset consists of 24 DNAm profiles of epidermal tissue which was extracted from non-irradiated sun-protected site on the upper buttocks. The samples were collected from a Caucasian female cohort, with younger individuals aged 19–28 (mean age = 23) and older individuals aged 65–81 (mean age = 71). These data are currently under embargo, and will be available on Array Express from 2027. See Shyne et al., (2024) for further details.

#### Epidermal and dermal lower outer and upper inner arm DNAm profiles – Vandiver dataset

We obtained 78 publicly accessible skin epigenetic profiles generated and described previously by Vandiver et al., (2015) from the Gene Expression Omnibus (GEO; accession: GSE52980). These data include Illumina 450k array profiles of 38 epidermis and 40 dermis biopsies from sun-protected and sun-exposed body sites in ten young and ten old volunteers.

### Facial skin swab dataset

#### Facial skin swab DNAm samples – Rotterdam Study dataset

This dataset consists of targeted DNA methylation amplicon sequencing (Ion Ampliseq) profiles for 44 nasolabial fold skin swabs of individuals in the Rotterdam Study cohort (Ikram et al. 2024)*.* As a lower cost targeted approach, 99 CpG sites were selected, including 25 sites used in the Endogenous Skin clock, 23 (of 24) sites used in the original Exogenous Skin clock (‘24-probe model’, see below), and 52 sites used in the Horvath Skin and Blood clock (Horvath et al. 2018). These 52 Skin and Blood clock sites were selected as the most age-informative CpGs of the Horvath Skin and Blood epigenetic clock based on their maximal contribution to the age estimation of the clock in 597 skin samples (GSE51954 (Vandiver et al. 2015), GSE77135 (Ivanov et al. 2016), GSE104471 (Yang et al. 2017), and GSE90124 (Roos et al. 2017)) using the approach of lasso-ridge regression iteration (see *Supplementary Methods* for further details). Donors were also assessed for facial photodamage severity based on the scales of Griffiths et al., (1992) and Ayer et al., (2018) by trained researchers (*Supplementary Methods*).

### Fibroblast datasets

Fibroblast cells were utilised from two collections carried out at Unilever pre-2005. Both studies collected 4mm full thickness punch biopsies from donors with informed written consent and ethical review. Due to the historical nature of the studies, further ethical review for the use of these anonymised fibroblasts for their study detailed here was sought and ethical approval was granted from an NHS REC (West Midlands - Black Country NHS REC), *Ageing in skin fibroblast cells*; reference: 16/WM/0041, IRAS project ID/approval form: 187622/901579/1/330.

#### Young and older donor matched protected exposed cultured fibroblasts – Fibroblast young old dataset

Fibroblast samples were extracted from the outer lower forearm and, donor matched, from the upper inner arm from female donors. Culturing and DNA profiling are detailed in *Supplementary Methods*. In-brief, cells were grown in two batches.

For the first batch, four old (age ≥ 71) and four young (age ≤ 33) donor cells were grown from lower outer arm and donor matched upper inner arm samples. Each of these sixteen fibroblast cultures was profiled in triplicate, and all 48 Illumina 450k array DNAm profiles were used in this study; data is available in ArrayExpress under E-MTAB-15156. These samples were used to test the performance of the epigenetic clocks.

#### Young and older exposed cultured fibroblasts – Fibroblast exposed dataset

For the second batch, outer lower forearm fibroblasts were grown from seven older (age ≥ 58) and six young (age ≤ 39) donors. In total, 13 were created and methylation data available in ArrayExpress under E-MTAB-15155. Because fibroblasts from six of these donors were also present in the previous batch above (Fibroblast young old dataset), they were not used from this second culture batch, leaving 7 Illumina 450k array DNAm profiles for training of the exogenous epigenetic clock (see below).

### Generation of the exogenous skin ageing epigenetic clock

#### Initial training

We first identified CpG sites on the Illumina 450k array which were differentially methylated between both young and old sun-exposed skin, but not between young and old sun-protected skin in 96 arm skin DNAm profiles. 2,259 CpG sites were identified as being associated with photoageing as previously described (Furtak *et al.,* 2026). Principal Component Analysis (PCA) identified CpG sites that contributed the most to differentiating participants by age in young to older photo-exposed sites. A total of 505 CpG sites were identified from this analysis and combined with a list of 156 CpG sites which were associated with a skin photodamage signature as described elsewhere (Furtak *et al.,* 2026). Of these CpG sites, 583 passed quality control checks in the Bormann dataset. We then trained a ridge regression model to predict age using these 583 sites from DNAm profiles of sun-exposed skin (the Bormann dataset and sun-exposed subset (N = 48) of the Furtak young old dataset) using the *glmnet* package in R (version 2.0-16) (Friedman, Hastie and Tibshirani 2010). We further subset the probes to select only those which were associated with increasing age in the subset of the Furtak young old dataset, and had a meaningful coefficient in the ridge regression model, identifying 308 CpG sites. Five elastic net regression models were trained to predict age on the same training sets, each with four-fold cross-validation. To minimise the number of CpG sites in the clock, the 28 sites which had non-zero coefficients in at least two of the five models were then used to train three new elastic net regression models. The four CpG sites which had coefficients of zero in all three of these models were then removed. The Exogenous Skin clock model was then created by averaging the model intercepts and model coefficients of the 24 remaining CpG sites from these three elastic net regression models (see *Supplementary Methods* for further details).

#### Re-training

To improve the performance of the Exogenous Skin clock on *in vitro* fibroblast epigenetic profiles, we trained a single new elastic net regression model using the original 583 CpG sites and three training datasets: the same two sun-exposed datasets used for the previous training, and an additional dataset of fibroblast DNAm profiles: the Fibroblast exposed dataset (Table 1).

Seven epigenetic profiles from the Fibroblast exposed dataset were included in the re-training, from four old (aged 58–82) and three young (aged 21–39) donors.

### Generation of the endogenous skin ageing epigenetic clock

To generate an Endogenous Skin Ageing epigenetic clock, we selected CpG sites by conducting a differential methylation analysis between young and old whole-skin samples from sun-protected sites (as described previously (Furtak et al., 2026)). After excluding CpG sites which were also differentially methylated between sun-protected and sun-exposed sites, we identified a total of 1,575 endogenous ageing CpG sites on the Illumina 450k array. PCA was performed to identify which of these sites contributed the greatest variance (PC1 loading > 0.03) to classifying young and old sun-protected skin (the sun-protected subset (N = 48) of the Furtak young old dataset) and identified 322 CpG sites for model training. Next, three elastic net regression models were trained to predict chronological age using DNAm profiles mostly from sun-protected skin (the Bormann dataset, the sun-protected subset of the Furtak young old dataset, a subset of the MuTHER dataset (N = 48; Table S1), and the Shyne dataset) (Table 3). In total, 114 sites had non-zero coefficients in at least one of these three models. Of these 114 sites, 107 were also present in the datasets for validation (Vandiver dataset, the publicly available samples from the MuTHER dataset not used in model training (N = 276)). Using the four training datasets (Table 3), the 107 CpG sites were used to train three elastic net regression models predicting age. Each of these models was trained using four-fold cross-validation, and 41 sites with non-zero coefficients in all three models were retained. To further optimise the model, only 25 CpG sites with model coefficients greater than 10% of the coefficient of the most impactful CpG site were retained. These were then used to train the Endogenous Skin clock model, using a ridge regression model which predicted chronological age in sun-protected skin DNAm profiles in the same four datasets.

### Gene expression association study of exogenous skin ageing acceleration in 364 whole-skin samples

To inform interpretation of the Exogenous Skin Ageing epigenetic clock, and to identify alterations in gene expression associated with accelerated exogenous ageing, we performed Gene Expression Association Studies (GEAS). To this end, we used data from 364 whole-skin biopsies from TwinsUK participants, which had DNAm profiles in the MuTHER dataset, and had also been profiled using RNA-sequencing (Shore et al. 2024; Buil et al. 2015). GEAS were performed for both Exogenous Skin clock age and age acceleration estimates, comparing the expression level of a total of 24,704 genes.

Trimmed Mean of M-values (TMM) normalised-Counts Per Million (CPM) gene expression values were fit to linear mixed models which included Exogenous Skin clock age/age acceleration, BMI, and technical covariates (mean GC content, batch, and median insert size) as fixed effects, and twin pair relatedness as random effects. This ‘fit’ model was then compared to a ‘null’ model, which was identical to the fit model but for omitting Exogenous Skin clock age/age acceleration as a fixed effect. ANOVA was used to obtain a p-value for the difference in the model fits (fit model vs null model). To account for multiple testing, p-values for each GEAS were adjusted using the q-value R package (version 2.34.0) (Storey et al. 2025), and a significant association was defined as q-value < 0.05.

To assess if the relative proportion of dermis and epidermis in these whole-skin epigenetic profiles influenced the results, a sensitivity analysis was performed for this GEAS, including the first principal component of the whole-skin DNAm data as a fixed effect covariate. Previously, we have shown that this PC captures the relative proportion of dermis to epidermis in this dataset (Shore et al. 2024). All other aspects of this sensitivity analysis were identical to the primary analysis.

### Gene expression changes between sun-exposed and sun-protected skin

To further validate the findings from the Exogenous Skin clock GEAS described above, we conducted a comparative analysis using an independent bulk RNA-seq dataset, generated using Lexogen QuantSeq 3' mRNA-Seq Kit, that identified differentially expressed genes between sun-exposed and sun-protected arm skin sites in 12 older individuals, aged 55–70 years (the ‘RNA-seq dataset’, available under E-MTAB-15291).

Raw count gene expression data was first normalized by estimating size factors and genes with fewer than five counts in less than three samples were excluded from downstream analysis. Principal component analysis (PCA) was then performed to assess clustering of samples and consequently detect potential outliers, but none were found. Next, differential expression analysis was performed using the DESEq2 package (version 1.46.0) (Love, Huber and Anders 2014), incorporating donor as a covariate to account for the paired sample design. Finally, correlation analysis was used to evaluate whether genes significantly associated with Exogenous Skin clock acceleration displayed a consistent direction of change in sun-exposed to sun-protected sites in this independent *in vivo* bulk RNA-seq dataset.

## Results

### Exogenous and endogenous Skin epigenetic clocks

Two epigenetic ageing clocks were built for skin; the first clock was an endogenous clock, which was trained using CpG sites that had significant young-to-old methylation changes in sun-protected sites, but did not have significant differential methylation between exposed and protected skin sites in a previous epigenetic study of arm skin (Furtak et al., 2026). This clock was trained to predict chronological age using data from whole-skin and epidermal samples (Table 3), and the final trained model used 25 CpG sites for age prediction (Table S2). The second clock was an exogenous clock that was trained using CpG sites initially selected in an arm skin study (Furtak et al., 2026) that had significant young-to-old methylation changes in sun-exposed but not sun-protected skin sites. This clock was trained to predict chronological age using sun-exposed whole-biopsy and epidermal samples (Table 1) and the final model used 24 CpG sites for age prediction (Table S3).

Of the 25 endogenous CpG sites, eleven were located in CpG islands, and four located in CpG shores (< 2 Kbp from a CpG island). The overall median distance of the 25 CpG sites to the nearest gene Transcription Start Site (TSS) was 679 bp (Table S2). The CpG site closest to a gene TSS was cg07922606, located 7 bp downstream of the TSS of the *HIST1H3E* gene, which encodes a protein in the Histone 3 family (Table S2).

In contrast, of the 24 exogenous CpG sites, none were located within CpG islands, eight were located in CpG shores, and three were located in CpG shelves (2–4 Kbp from a CpG island). The median distance to the nearest gene TSS was 6,791 bp (Table S3). The CpG site closest to a gene TSS was cg14250984, located 276 bp upstream of the TSS of the *EEF1G* gene, which encodes a translation elongation factor (Table S3).

Next, to validate the Endogenous and Exogenous Skin Ageing epigenetic clocks, we applied both clock algorithms, in addition to the Horvath Skin and Blood epigenetic clock algorithm (Horvath et al. 2018), to three independent, previously published datasets.

### Age prediction in sun-protected versus sun-exposed arm skin samples (Vandiver dataset)

**Fig. 1:**
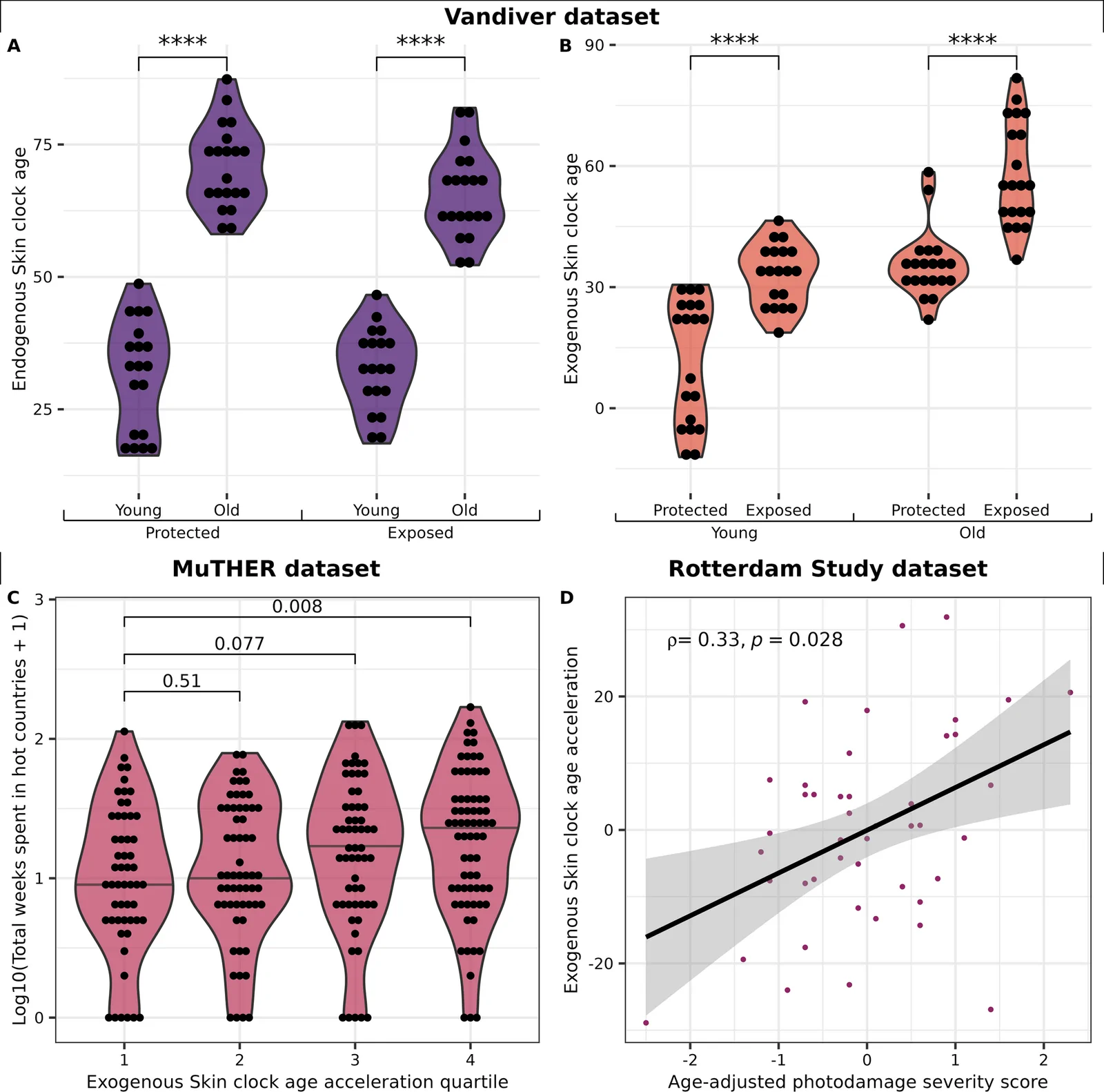
Validation of the Endogenous and Exogenous Skin Ageing epigenetic clocks. **A** Endogenous Skin clock age estimates in DNAm profiles of young and old, sun-protected and sun-exposed skin. Age predictions from dermal and epidermal samples are shown together. **B** Exogenous Skin clock age estimates in DNAm profiles of young and old, sun-protected and sun-exposed skin. Age predictions from dermal and epidermal samples are shown together, but can be observed separately in Fig. 2A. **C** Differences in the Exogenous Skin clock age acceleration estimates (grouped by quartile) with the (log transformed) self-reported number of weeks spent in hot countries. **D** Correlation between age-adjusted photodamage severity score and Exogenous Skin clock age acceleration estimates

First, we generated the epigenetic clock age and age acceleration estimates in 78 publicly available DNAm profiles generated by Vandiver et al., (2015) from epidermal and dermal skin samples. The ‘Skin and Blood’ and Endogenous Skin epigenetic clocks generated significantly older age estimates for profiles in the ‘old’ group compared to the ‘young’ group, as expected (Fig. 1A and S1), with a median age error (MAE) of 2.9 and 7.3 years, respectively (Fig. S2). We also observed no significant difference between epigenetic age estimates for sun-exposed samples compared to sun-protected samples (ANOVA *p* = 0.66 and *p* = 1.00, respectively; Fig. S3), suggesting that these clocks are unlikely to capture the effects of exogenous skin ageing.

In contrast, the Exogenous Skin clock generated age estimates which were significantly older in the sun-exposed samples, compared to the sun-protected samples, in both the young and old age groups (Fig. 1B, p = 9.6×10^−8^). The Exogenous Skin clock age estimates were also more accurate in predicting chronological age in sun-exposed skin sites, with an MAE of 9.2 years in these sites, compared to 31.0 years in sun-protected skin sites (Fig. S4). In addition, the Exogenous Skin clock age estimates for the old sun-exposed samples were significantly older than those for the young sun-exposed samples (Fig. 1B, p = 1.4×10^−8^). This suggests that the Exogenous Skin epigenetic clock captures biological effects which accumulate mainly in sun-exposed sites, although intrinsic body site differences (such as cell developmental origin) could also influence these results.

### Age prediction and sun-seeking behaviour in abdominal skin samples (MuTHER dataset)

Next, to investigate performance in a different body site and with sun-exposure related lifestyle factors, we applied the Endogenous and Exogenous Skin Ageing epigenetic clocks to 414 whole-skin DNAm profiles from older female twins who were predominantly free from major disease (Nica et al. 2011; Roos et al. 2017; Shore et al. 2024)*.* These methylome data were generated from skin biopsies from a relatively sun-protected region adjacent to the umbilicum, as part of the TwinsUK MuTHER project (Nica et al. 2011; Verdi et al. 2019).

The Endogenous Skin clock age estimates showed a high accuracy in predicting chronological age (MAE = 4.21 years, rho = 0.76, *p* < 2.2×10^−16^; Fig. S5 (excluding 48 profiles used in Endogenous Skin clock training)), as did the Skin and Blood clock (MAE = 4.7 years, rho = 0.87, *p* < 2.2×10^−16^; Fig. S5). As with the Vandiver dataset, the Exogenous Skin clock was a poorer predictor of chronological age than both other clocks (MAE = 13.4 years, rho = 0.39, *p* < 2.2×10^−16^; Fig. S5).

Of the 414 individuals for whom whole-skin DNAm profiles were available, 242 had previously collected questionnaire data on sun-seeking behaviour (Sanna et al. 2021). The sun-seeking data was collected ~10 years prior to the whole-skin biopsy collection, although sun-seeking behaviour has been shown to be relatively stable over time (Sanna et al. 2021), and we assume that it captures, to an extent, lifetime sun-exposure of abdominal skin. Indeed, we observed a significant positive correlation between Exogenous Skin Ageing epigenetic age acceleration and sun-seeking behaviour (Rho = 0.184, *p* = 0.004, Fig. 1C). However, we did not see this correlation for either the Endogenous Skin clock or the Skin and Blood clocks (Fig. S6).

These findings indicate that older ages in sun-exposed sites are influenced by sun-exposure and not just body site differences, at least in whole-skin tissue biopsies.

### Age predictions in non-invasive facial skin swabs (Rotterdam Study dataset)

Next, we investigated whether the Exogenous and Endogenous Skin clocks associated with photodamage grading are robust when estimated using non-Illumina DNAm microarray methylation profiles. We assessed the applicability of these clocks using DNAm profiles of 44 skin swabs from the nasolabial fold of participants from the Rotterdam Study dataset, generated using targeted bisulphite amplicon sequencing using Ion Ampliseq technology of 99 CpG sites (*Supplementary Methods*) (Ikram et al. 2024).

Both the resulting Exogenous and Endogenous Skin clock ages significantly correlated with chronological age in this sample, as did the (52 CpG site version) Skin and Blood clock (MAE = 12.5, 6.05, and 4.45, respectively; Fig. S7). Additionally, the age acceleration residuals of the Exogenous Skin clock correlated with photodamage severity scores (rho = 0.33, *p* = 0.028, Fig. 1D). No such significant correlations were observed with the age acceleration estimates from the Endogenous Skin clock (rho = 0.08, *p* = 0.59), or from the Skin and Blood clock (rho = − 0.13, p = 0.42) (Fig. S8).

### Age predictions in dermal skin and *in vitro* dermal fibroblast DNAm profiles

**Fig. 2:**
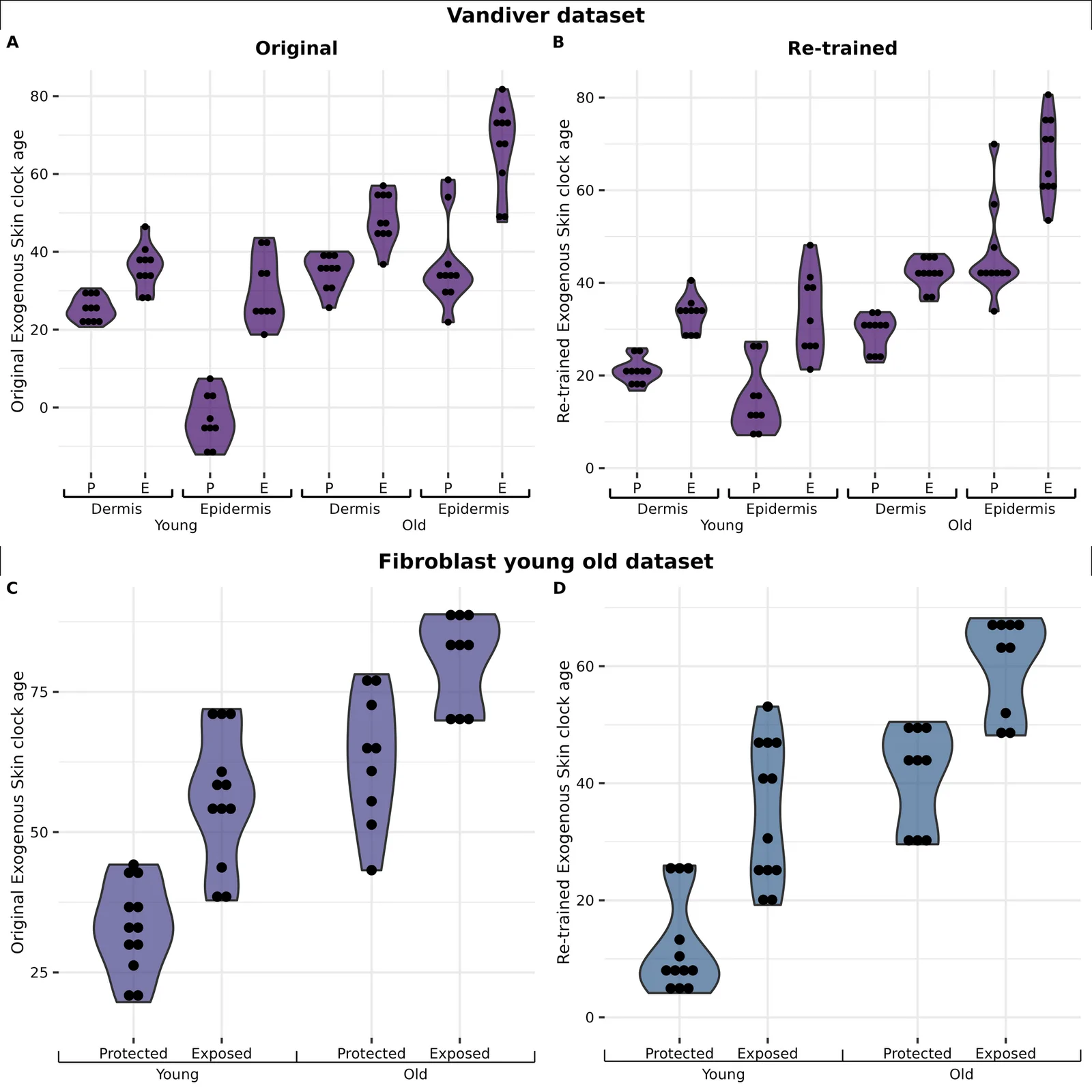
Performance of the original and re-trained Exogenous Skin clocks on DNAm profiles from:** A** and **B** dermis and epidermis samples from sun-protected (P) and sun-exposed (E) sites in young and old donors (Vandiver dataset), and **C** and **D** *in vitro* dermal fibroblast samples from sun-protected and sun-exposed skin sites from young and old donors (fibroblast young old dataset)

The Exogenous Skin clock was trained using DNAm profiles generated from epidermal and whole-skin samples. To examine its performance in dermal samples we tested age prediction in the Vandiver dermal dataset (Vandiver et al. 2015). Sun-exposed dermal tissues had more similar ages to sun-protected dermal tissues compared to epidermal age predictions (Fig. 2A), indicating it does not distinguish dermal exogenous changes as well as epidermal exogenous changes.

We next tested their performance using *in-vitro* samples and applied the clock to dermal fibroblast derived data from sun-protected and (donor matched) sun-exposed sites from four young and four old donors (the fibroblast young old dataset). The Exogenous Skin Ageing epigenetic clock estimated younger ages in the fibroblasts from sun-protected sites compared to those from sun-exposed sites (ANOVA *p* = 0.0003, Fig. 2C). The clock also distinguished between fibroblasts from young sun-exposed sites and those from older sun-exposed sites, respectively (ANOVA *p* = 3.5×10^−5^, Fig. 2C). However, the MAE of the exogenous age for fibroblasts from sun-exposed sites in young donors was 13.5 years (Fig. 2C), suggesting that the clock is not as well calibrated to donor age estimation for fibroblasts as to donor age estimation for whole-biopsy skin and epidermal samples.

### Improving clock performance in dermal fibroblast DNAm profiles

To improve the accuracy of the Exogenous Skin clock in DNAm profiles of *in vitro* dermal fibroblast cultures and dermis biopsies, we re-trained the clock. Re-training was performed similarly to the initial training of the Exogenous Skin clock, including using the same list of 583 candidate CpG sites (see *Methods*). However, in addition to using the training datasets used to train the first version of the epigenetic clock (the Bormann dataset and sun-exposed subset of the Furtak young old dataset; Table 1), we also included an additional seven DNAm fibroblast profiles from sun-exposed sites from three young and four old donors (fibroblast exposed dataset; Table 1) in the training data of this second version of the Exogenous Skin epigenetic clock.

The re-trained Exogenous Skin clock included a total of 42 CpG sites, eleven of which were also included in the original version of the clock. Similarly to the original Exogenous Skin clock, most CpG sites were not located in CpG Islands (8/42 sites). The median distance to the nearest gene TSS of 7621.5 bp, and the CpG site closest to a gene TSS was cg19009417, located 104 bp upstream of the *RGR* gene (Table S4).

After re-training, there was an improvement in the MAE of the re-trained clock in the fibroblast young old dataset, with MAE values of 10.0 years for the fibroblasts from exposed sites (Fig. 2D). The re-trained clock also continued to distinguish between sun-exposed and sun-protected profiles in both old and young donors (Fig. 2D).

We also observed an improvement of the accuracy of the Exogenous Skin clock in the Vandiver dataset, with an overall MAE of 13.6 years for the re-trained clock (vs 17.7 years for the original clock). Furthermore, we observed a greater distinction between sun-protected and sun-exposed dermis profiles (Fig. 2B).

Finally, we found similar ages and findings when applying the re-trained Exogenous Skin clock on arm epidermal samples (Vandiver dataset) and abdominal samples (TwinsUK MuTHER dataset) as the original exogenous clock (Figs. 2B, S5D, S6C and S8–S10). Further analyses in this study exclusively utilised the re-trained version of the Exogenous Skin clock.

### Exogeneous epigenetic age associations with gene expression

Next, to further explore the molecular biology changes associated with these clocks, we performed Gene Expression Association Studies (GEAS) of Endogenous Skin clock age acceleration, Skin and Blood clock age acceleration and Exogenous Skin clock age acceleration, to identify potential changes in gene function with epigenetic ageing. These analyses were carried out using 364 whole-skin samples from the MuTHER cohort which had undergone both epigenetic and transcriptomic profiling (Buil et al. 2015; Roos et al. 2017; Shore et al. 2024).

**Fig. 3:**
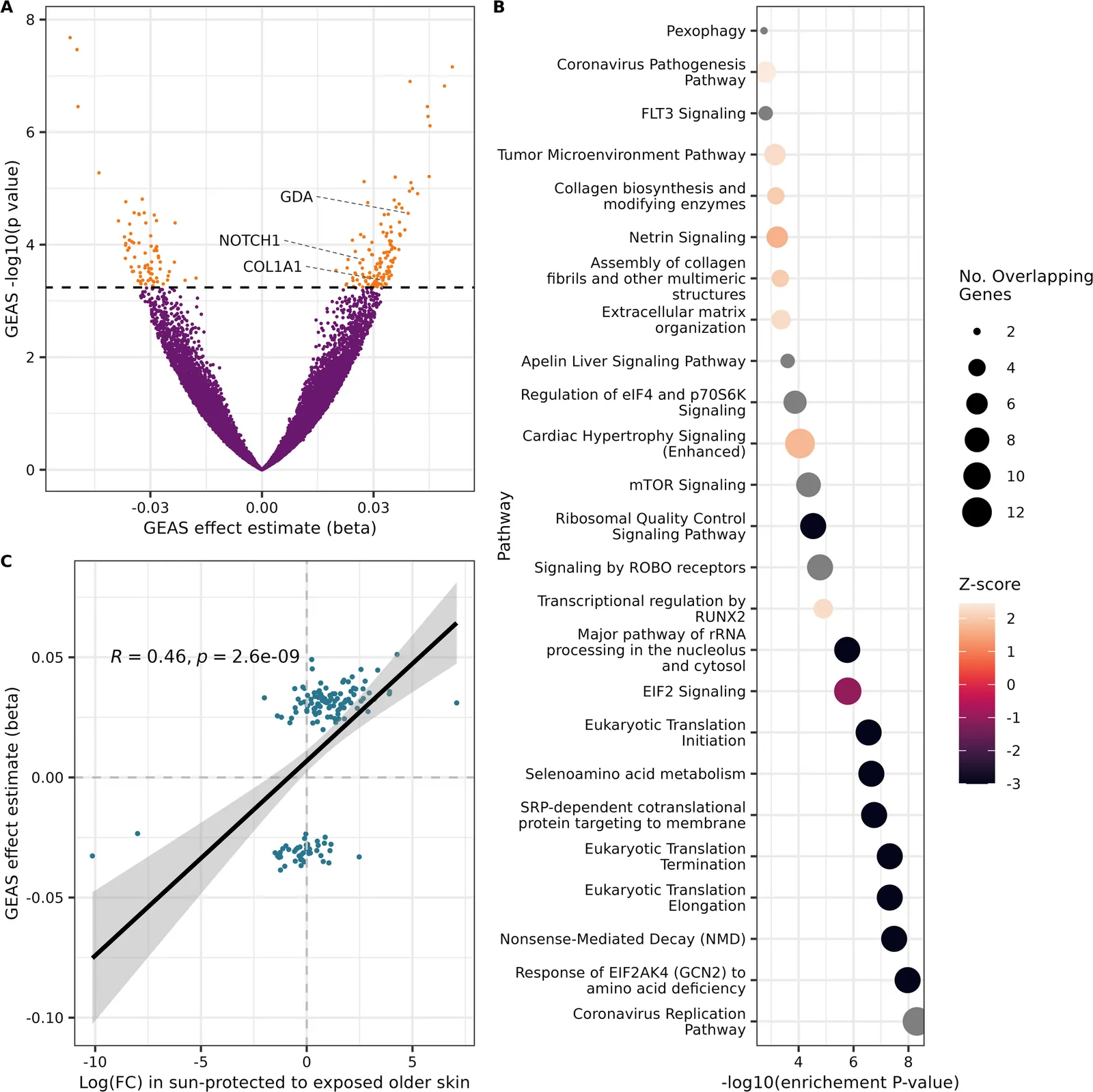
Gene expression analysis of mRNAs associated with the exogenous clock. **A** Effect estimate vs –log10(*p*-value) of the GEAS of Exogenous Skin clock acceleration in 364 whole-skin samples. **B** The 25 canonical pathways most significantly enriched for genes associated with Exogenous Skin clock acceleration. The full results of these analyses are in Table S6. **C** Correlation between effect estimates from the GEAS of Exogenous Skin clock acceleration, and the log(fold-change) values from a differential expression analysis between sun-exposed whole-skin relative to sun-protected whole skin, for 134 overlapping genes

No genes had significantly associated expression levels with the Endogenous Skin clock or the Skin and Blood clock age acceleration, but 202 gene mRNA levels (0.8%) were significantly associated with Exogenous Skin epigenetic age acceleration (q-value < 0.05, Table S5). Of these, 128 showed increased expression with accelerated Exogenous Skin Age, while 74 showed decreased expression (Fig. 3A; Table S5). Of note, a significant increase in *COL1A1, EDN3 and GDA gene* expression with increased Exogenous Skin clock acceleration was observed (beta = 0.033 and *p* = 0.0004, beta = 0.05 and *p* = 6.9×10^−8^, beta = 0.039 and *p* = 2.8×10^−5^ respectively; Fig. 3A, Table S5).

Canonical pathway enrichment analysis of these 202 genes showed that 25 pathways (4.0%) were significantly enriched at FDR < 0.05, including a predicted decrease in the activity of the EIF2 signalling pathway (Fig. 3B, Table S6).

Finally, a sensitivity analysis of this GEAS was performed, which adjusted for the relative proportions of dermis and epidermis in this whole-skin dataset (see *Methods*). The effect sizes and log-transformed p-values of this sensitivity analysis were highly correlated with the original GEAS (R = 0.997, *p* < 2.2×10^−16^ and R = 0.995, *p* < 2.2×10^−16^, respectively; Fig. S12), and of the 202 significant genes in the primary analysis, 97 remained significant at an FDR-corrected significance threshold (q-value < 0.05), and all 202 were nominally significant at a threshold of *p* < 0.005 in this sensitivity analysis (Table S8). This suggests that in this dataset, the relative proportions of epidermis and dermis in each whole-skin profile are not substantially confounding the findings.

### Validation of the exogenous clock gene expression signature

To validate the findings of our GEAS of Exogenous Skin Age clock acceleration, we performed differential gene expression analyses using an independent RNAseq dataset. Specifically, we assessed the impact of sun-exposure on gene expression in 24 bulk RNA-seq profiles of sun-protected and sun-exposed whole-biopsy arm skin from twelve older donors (aged 55–70).

In total, 134 of the 202 genes which were significantly associated with Exogenous Skin Age acceleration and passed quality checks in this independent dataset (Table S6). While only seven of these 134 genes were significantly differentially expressed in this analysis (FDR < 0.05; Table S6), the log(FC) of all 134 genes correlated strongly and positively with the effect estimates from the Exogenous Skin Age acceleration GEAS (Rho = 0.46, p= 2.6x10^-9), validating the previous RNA findings (Fig. 3C).

Seven significant genes were observed in both the GEAS of Exogenous Skin age acceleration and in the differential exposed to protected expression analysis. These included *COL1A1*, *SFRP2*, *CDH4*, *COL14A1*, *CDH5*, *SPARC*, and *THY1* (Tables S5, S7). For all of them, the direction of effect observed for accelerated Exogenous Skin clock age was the same as that for sun-exposed skin relative to sun-protected skin (Tables S5, S7; Fig. S11).

## Discussion

In this study we described the construction and validation of two skin-specific epigenetic ageing clocks. We found that the Endogenous Skin clock strongly correlates with chronological age, while the Exogenous Skin clock is associated with both chronological age and sun exposure in epigenetic profiles of both *in vivo* and *in vitro* skin tissues and cells. Furthermore, we demonstrated the applicability of the Exogeneous Skin Ageing clock in predicting biological age from non-invasive skin samples obtained via nasolabial fold swabs and observed a significant positive correlation with photodamage severity. Lastly, we showed that the Exogenous Skin clock is associated with changes in the whole-skin transcriptome, including in several genes previously linked to exogenous skin ageing, highlighting the potential of this clock to further understanding of exogenous skin ageing.

DNAm changes have been demonstrated to be one of the most accurate markers of not only chronological age from biological samples, but also of how well someone is ageing considering their chronological age – i.e., biological age (Horvath 2013; Lu et al. 2019; Levine et al. 2018; Hannum et al. 2013). For skin, ageing is driven by environmental factors and their interactions with internal factors such as genetic variation (Jeremian et al. 2024). Photo-ageing is one such example of these interactions and is one of the strongest drivers of skin ageing, particularly in exposed skin sites of individuals with lighter skin colour (D’Arino et al. 2023; Ibrahim et al. 2023; Jeremian et al. 2024). However, current epigenetic clocks do not distinguish between photo-ageing from other types of skin ageing. Here, we demonstrate that epigenetic clocks can be tailored to different aspects of skin ageing. By only using DNAm levels of CpG sites that change in sun-exposed sites rather than in sun-protected sites, we were able to build a clock that predicted older ages in sun-exposed sites compared to protected sites in an independent set of samples. In addition, the difference between the epigenetically predicted age and the donor’s true chronological age was correlated with sun-seeking behaviour and photodamage grading in non-invasive samples. Hence, although the associations only explained some of the variation in sun-seeking behaviour or photodamage, these results confirm that an epigenetic clock can reflect exogenous ageing in skin to a certain degree.

The Exogenous Skin clock acceleration association with sun-seeking behaviour was evident despite this phenotype being collected ~10 years prior to the whole-skin biopsies used for epigenetic profile generation. Given the statistically significant, albeit weak, correlation between this phenotype and the Exogenous Skin clock acceleration it is unclear whether a more accurate measure of the participants’ abdomen sun-exposure would strength this association and, thus, further validation is required. Similarly, the photodamage phenotype in the Rotterdam Study dataset was generated across only 4 grades, the DNAm data was generated from facial swab samples (representing desquamated keratinocytes) and was generated using a different DNAm measurement technique (DNA methylation amplicon sequencing) to those used in the training datasets. Indeed, the skin swabs represent methylation differences with age in desquamated keratinocyte-derived DNA, limiting its relevance to more broad-based skin ageing phenomena (such as dermal changes) likely evident in the full skin training dataset ( in these samples. Taken together these factors should have limited the extent the Exogenous Skin clock acceleration and age-adjusted photodamage severity score correlated. However, the inclusion of facial swab samples and amplicon sequence data in the training datasets should be tested to determine its true effect on the correlation with photodamage grading in future studies.

The Endogenous Skin clock was built to solely focus on ageing occurring in sun-protected sites. Indeed, this clock did not predict older ages in sun-exposed sites, nor did its age acceleration reflect sun-seeking behaviour or photodamage grading. Hence, the combination of the Exogenous Skin clock and the Endogenous Skin clock or the Skin and Blood clock can distinguish between these two types of ageing in skin samples and help disentangle the complex interplays occurring in human skin ageing.

Skin *in-vitro* models of ageing are useful in, for example, not only understanding how stressors cause damage to skin cells but also in helping translate interventions from the laboratory to the clinic. To understand whether the exogenous clocks could be used on fibroblast *in-vitro* samples, we tested its performance in both dermal and fibroblast samples. Although significant differences were observed, the Exogenous Skin clock was less able to distinguish between sun-exposed and sun-protected dermal samples than epidermal and whole-skin samples. We found that re-training of the exogenous clock including fibroblast samples improved its performance on dermal and cultured fibroblast samples whilst not degrading its performance in epidermal and whole skin samples. This highlights the fact that epigenetic clocks can be continuously updated to improve the performance or to help with applicability to differing types of samples. For skin, this is particularly relevant, as the dermis and epidermis are comprised of different cell types, have dramatically different cell-turnover rates, and show different responses to photoageing (Vandiver et al. 2015). In addition, the inclusion of CpG sites for age predictions that are different between sun-exposed and sun-protected samples could allow for influences from intrinsic body-sites differences rather than just photoageing differences. However, these CpG sites were also selected on the condition they significantly changed in a similar manner with age within the same sun-exposed body site and, therefore, this should have enriched for CpG sites that are different between sun-exposed to sun-protected body-sites due to photoageing rather than intrinsic body site differences. However, further validation to fully rule out such intrinsic body site influences on the Exogenous Skin age predictions is warranted.

While re-training the Exogenous Skin clock appeared to improve performance, the resulting changes of this re-training may be difficult to track. This is particularly important when applying the Exogenous Skin clock to other datasets. For example, we believe the retrained, second version of the Exogenous Skin clock to be the most appropriate version of the Exogenous skin clock for use in dermal and fibroblast samples. However, we were only able to validate the performance of the earlier, original version of the clock on non-invasive skin swab samples, as the methylation profiles of these samples lacked data on most of the CpG sites in the retrained Exogenous Skin clock. Subsequently, the performance of the retrained version of the Exogenous Skin clock in epigenetic profiles from non-invasive skin swab samples remained unvalidated. In general, compared to blood, available DNAm skin sample datasets are lacking in their coverage of different body sites and type of samples (for example epidermal samples can be obtained by suction blisters, tape strips, swabs or biopsy shaves). Indeed, whilst the correlation of the Exogenous skin clock to facial photodamage grading in skin swabs facilitates use of non-invasive sampling in future studies, its relevance to and association with deeper skin changes is unknown. Further datasets and comparisons covering these types of variables are required to get a better understanding of the performance of different skin clocks across clinical and *in-vitro* samples, and between different body sites, sample type and DNAm measurement platforms and technologies.

We also investigated what biological mechanisms the epigenetic clocks could be reflecting using age acceleration associations with mRNA levels. Only the (re-trained) Exogenous Skin clock gave significant associations, possibly as it is likely dominated by photodamage effects, whereas the Endogenous Skin and Skin and Blood clocks are likely reflecting a greater range of ageing factors and mechanisms (e.g. nutrition, smoking, stress etc). Therefore, in the latter two clocks, each individual factor is a smaller aspect of the age acceleration and, thus, of diluted influence and greater difficulty to detect. The significant correlation between RNA levels associated with Exogenous Skin clock acceleration, and sun-exposed to sun-protected RNA differences in an independent study confirm that the Exogenous Skin clock is capturing biological and molecular changes associated with exogenous skin ageing independently of chronological age.

Pathways enriched with the Exogenous Skin clock age acceleration included the EIF2 signalling pathway which has previously been shown to promote protective molecular changes in the epidermis in response to UV-A and UV-B radiation exposure (Collier, Wek and Spandau 2015). In addition, the MTOR signalling pathway was enriched which, although there was no clear direction of change, has previously been implicated in UV-induced ageing from both UV-A and UV-B radiation (Chen et al. 2022). Finally, activity of the “Tumor Microenvironment Pathway” was predicted to increase with accelerated Exogenous Skin clock age, which is consistent with studies which find an increased risk of skin cancers (including melanoma) with increased sun exposure (and thus exogenous ageing) (Chen et al. 2022; D’Arino et al. 2023). These findings indicate that the Exogenous Skin clock is capturing, to a degree, the effects of photoageing. Further investigation, through single cell techniques, into whether these associations are due to cell-type differences within skin samples or gene expression changes within cells, is now required to better understand the biological mechanisms driving these associations.

One notable finding in the individual RNA results was an increase in *COL1A1* expression with accelerated exogenous clock age. Previous studies examining the dermal proteome identified a decrease of Type I Collagen protein in photo-aged skin (Liu et al. 2024)*;* thus, it might be expected that individuals with accelerated exogenous ageing would have decreased *COL1A1* expression, contrary to our findings. However, a recent study examining changes in *COL1A1* transcript expression (rather than Type I Collagen) in young and old sun-protected and sun-exposed skin identified a pattern which corroborates our finding (Yan et al. 2024)*.* Specifically, the study found that *COL1A1* expression showed a slow but consistent decrease over time in sun-protected skin, and a small and initially rapid decrease which plateaued in early life in sun-exposed skin. Hence, at older age, *COL1A1* mRNA had declined less in sun-exposed samples than sun-protected samples. Because we performed our GEAS using epigenetic profiles of sun-protected whole-skin from older individuals (mean age = 59), the decrease in *COL1A1* expression caused by endogenous skin ageing likely outweighs the early life decrease caused by exogenous skin ageing. Thus, although COL1A1 does go down slightly with age, is reduces a lot less than with endogenous ageing which suggests UV exposure slows the rate of decline. Whether this is also the case at the protein level needs confirming along with further validation of how UV exposure might be slowing the rate of *COL1A1* mRNA decline with age.

While we have shown here that these novel epigenetic clocks perform well under a variety of experimental conditions, there are several limitations to these clocks. First, while we compared the performance of these clocks with the Skin and Blood epigenetic clock (Horvath et al. 2018), we did not compare them with the only other previously published skin ageing clock to date, developed by Boroni et al., (2020). This was because Boroni et al., (2020) developed their clock using data from Vandiver et al., (2015) and a subset of the MuTHER dataset (GSE90124) (Roos et al. 2017), which we also used for training and validation of the Endogenous and Exogenous Skin clocks. Second, while we validated the performance of these clocks under several different experimental conditions, a lack of publicly available data meant that we could not validate the performance of these clocks in certain skin epigenetic profiling contexts. Most notably, we did not apply these clocks to skin epigenetic profiles generated using whole-genome bisulphite sequencing or long-read sequencing technologies, which are increasingly favoured methods for epigenetic profiling. Finally, for the exogenous clock to be more widely utilised, further validation of its link to photodamage is required due to the relatively low, albeit significant, correlations with sun-seeking behaviour questionnaire and photodamage grading data.

The development of the Endogenous and Exogenous Skin clocks opens several avenues for future research and has several promising applications. For example, researchers developing anti-skin ageing therapeutics and interventions could use these two clocks to distinguish the impact of such therapeutics specifically on endogenous and exogenous skin ageing, expanding biological and clinical insight. Another possible application is the assessment of a patient’s endogenous and exogenous skin age, improving personalised skin health recommendations and treatment. Further validation and comparison to other skin clocks, though, is required to better understand the utility of these clocks for different research objectives.

In conclusion, we presented and validated the Endogenous and Exogenous Skin Ageing epigenetic clocks, which we believe will be an effective tool for future skin ageing researchers, and will contribute towards the development of more effective, at least based on DNAm ageing changes, and even personalised skin ageing therapeutics.

## Electronic supplementary material

Below is the link to the electronic supplementary material.

Supplementary file 1 (DOCX 7301 kb)

Supplementary file 1 (DOCX 39 kb)

Supplementary file 4 (XLSX 4516 kb)

## Data Availability

The Bormann, Furtak Young-Old, Fibroblast Exposed, and Fibroblast Young-Old datasets are available on ArrayExpress under the accession numbers E-MTAB-4385, E-MTAB-14619, E-MTAB-15155, and E-MTAB-15156, respectively. The Shyne dataset is available in array express from 2027, currently under embargo. The Vandiver dataset and the subset of the MuTHER DNAm dataset (the latter used to train the Endogenous Skin clock) are available on the Gene Expression Omnibus (GEO) under accession numbers GSE52980 and GSE90124, respectively. The full MuTHER DNAm dataset is available on the European Genome-Phenome Archive (EGA) under accession number EGAS00001007816. Due to restrictions based on privacy regulations and informed consent of the participants, data from the Rotterdam Study cannot be made freely available in a public repository. However, data may be obtained upon request. Requests for data from the Rotterdam Study should be directed to the management team of the Rotterdam Study (datamanagement.ergo@erasmusmc.nl), which has a protocol for approving data requests.
