# Supplementary material for: Epigenetic clocks can differentiate between exogenous and endogenous skin ageing
Source: Clin Epigenetics. 2026 Sep 11;18:168. doi: 10.1186/s13148-026-02230-w (PMC13591841; doi:10.1186/s13148-026-02230-w)
Supplement: Supplementary file 1 [file 13148_2026_2230_MOESM1_ESM.docx]

**Figure S1: Age estimates from the Horvath Skin and Blood epigenetic clock in DNAm profiles from the Vandiver dataset.**

**Figure S2: Chronological age of profiles in the Vandiver dataset (x-axes), compared with epigenetic clock age estimates from** **A**) the Horvath Skin and Blood epigenetic clock, and **B**) the Endogenous Skin epigenetic clock.

**Figure S3: Skin-site sun-exposure status in the Vandiver dataset, compared with age estimates from** **A**) the Horvath Skin and Blood clock, and **B**) the Endogenous Skin clock.

**Figure S4: Chronological age vs Exogenous Skin clock age (original, 24 CpG site clock version) in Vandiver dataset profiles of skin from** **A**) sun-protected sites, and **B**) sun-exposed sites. The dashed line represents the X=Y line, corresponding to a mean age error of 0.

**Figure S5: Chronological age of whole-skin DNAm profiles in the MuTHER dataset, compared with epigenetic clock estimates from A**) the Horvath Skin and Blood clock, **B**) the Endogenous Skin clock, **C**) the original (24 CpG site) version of the Exogenous Skin clock, and **D**) the re-trained (42 CpG site) version of the Exogenous Skin clock.

**Figure S6**: **Comparison of self-reported sun-seeking behaviour of 242 individuals who also provided whole-skin biopsies which were epigenetically profiles in the MuTHER study, with the quartile of age acceleration calculated using the** **A**) Horvath Skin and Blood clock, **B**) the Endogenous Skin clock, and **C**) the re-trained (42 CpG site) version of the Exogenous Skin clock.

**Figure S7: Chronological age in targeted bisulphite amplicon sequencing epigenetic profiles of facial skin-swabs from the Rotterdam Study dataset, compared with epigenetic age estimates from the** **A**) 52 CpG site version of the Horvath Skin and Blood clock, **B**) Endogenous Skin clock, and **C**) original (24 CpG site) version of the Exogenous Skin clock.

**Figure S8: Age-adjusted photodamage severity score, as assessed by trained researchers (see *Supplementary Methods*) for individuals with targeted facial skin-swab epigenetic profiles in the Rotterdam Study dataset, compared with epigenetic age acceleration estimates calculated using the** **A**) Horvath Skin and Blood clock, and **B**) the Endogenous Skin clock.

**Figure S9: Comparison of chronological age of epigenetic profiles in the Vandiver dataset, with epigenetic age calculated using the re-trained (42 CpG site) version of the Exogenous Skin clock, subset into profiles from A**) sun-protected skin sites, and **B**) sun-exposed skin sites.

**Figure S10: Comparison of the original (24 CpG site), and re-trained (42 CpG site) versions of the Exogenous Skin clock in skin epigenetic profiles from A**) the Vandiver dataset, and **B**) the MuTHER dataset.

**Figure S11: Comparison of Log Fold-Change of gene expression between sun-protected and donor-matched sun-exposed older skin samples, with effect estimates of the same genes in a Gene-Expression Association study of age acceleration estimated using the re-trained (42 CpG site) version of the Exogenous Skin clock in an independent dataset.** Only the seven genes which had significantly different expression between sun-exposed and sun-protected skin sites and were significantly associated with Exogenous Skin clock age acceleration, are shown.

**Figure S12: Correlation of A) effect sizes and B) -log10(p-values) from the primary GEAS of Exogenous Skin clock acceleration (x-axes), and the sensitivity, tissue-type adjusted GEAS of Exogenous Skin clock acceleration (y-axes).**
