# Supplementary material for: Epigenetic clocks can differentiate between exogenous and endogenous skin ageing
Source: Clin Epigenetics. 2026 Sep 11;18:168. doi: 10.1186/s13148-026-02230-w (PMC13591841; doi:10.1186/s13148-026-02230-w)
Supplement: Supplementary file 2 [file 13148_2026_2230_MOESM2_ESM.docx]

**Supplementary Methods**

**Selection of CpG sites to profile using Methylation Ampliseq technology in the Rotterdam Study**

To identify the most age-predictive markers in the Horvath Skin and Blood clock (Horvath et al., 2018), a lasso-ridge regression iteration was performed, resulting in the selection of 52 most informative CpG sites. To this end, 597 publicly available skin DNAm profiles were obtained (GSE51954, GSE77135, GSE104471, and GSE90124). The lasso-ridge regression iteration process consisted of the following steps:1) Only CpGs showing a statistically significant correlation (p<0.05) between chronological age and their beta-values were considered for further analysis. 2) A lasso regression model was applied to select the 52 most informative CpG sites from the filtered set. 3) A ridge regression model was used to evaluate the contribution of each of the 52 CpGs to MAE reduction. MAE reduction was estimated using a 5-fold cross-validation repeated 10 times. 4) In each iteration, the CpG with the least contribution to MAE reduction was excluded. The process returned to the second step, where the lasso regression model was reapplied to re-select 52 CpGs from all available CpGs after exclusion. This iterative process continued until all re-selected 52 CpGs contributed at least 0.005 years to MAE reduction.

To demonstrate the effectiveness of the method, we compare the predictive performance of different CpG selection methods, including lasso, lasso-linear (linear regression on CpGs selected by lasso), lasso-ridge (ridge regression on CpGs selected by lasso), and lasso-ridge iteration. A 5-fold cross-validation was performed on the full dataset. Specifically, the dataset was split into training (80%) and testing (20%) sets. Different CpG selection methods were applied to the training set to select 52 CpGs, and their predictive performance was evaluated on the testing set. This 5-fold cross-validation process was repeated 50 times to generate average performance, which were used to determine the optimal CpG selection method. The lasso-ridge regression iteration method demonstrated the best predictive performance among the tested methods (mean MAE=3.86 yr, compared with others ranged from 4.2 to 4.52 yr); thus, it was applied to the full dataset to select the final set of 52 CpGs.

**Phenotyping the severity of facial photodamage of Rotterdam Study participants**

Within the Rotterdam Study, participants also have their facial photograph taken during their on-site visit to the Rotterdam Study research centre in Ommoord, Rotterdam. Photodamage/-ageing was evaluated using photo-numeric grading scales for hypertrophic and atrophic photodamage based on the scales of Griffiths et al. (1992) and Ayer et al. (2018). Both photo-numeric grading scales consisted of a nine-point grading system. First, photographs of participants were evaluated and scored as either having hypertrophic or atrophic facial photodamage. Secondly, using the respective photo-numeric grading scale per subtype of photodamage, the photographs were scored on the severity of facial photodamage with the following classification: 0) no photodamage, 2) mild photodamage, 4) moderate photodamage, 6) moderate to severe photodamage, and 8) severe photodamage. The classification was recoded to 2 being 1, 4 being 2, 6 being 3, and 8 being 4 for more intuitive interpretation. Two researchers (C.E. and E.B.) underwent training before grading all photographs individually. Disagreements were discussed and resolved by agreement between the two researchers; when no agreement was reached, a third independent researcher (P.V.) was consulted.

**Initial training of the Exogenous Skin Ageing clock**

To identify probes that can predict age and reflect the effects of exogenous aging on the skin, the extrinsic age prediction model was trained using methylation data from sun-exposed skin sites. Probes that showed a consistent direction of change with increasing age and sun exposure were specifically selected, thereby ensuring that the model accurately captures the impact of exogenous aging on skin.

The probe selection process was initiated by conducting a differential methylation analysis between young and old samples from sun-exposed sites (Furtak protected dataset, n=48, E-MTAB-14619), which identified 10,649 CpG sites that were differentially methylated between young and old samples (Furtak et al. (under review)). These sites were further subset by removing probes that were not significantly different between sun-protected compared to sun-exposed body sites in young and old samples, thereby ensuring the removal of endogenous ageing changes not associated with exogenous ageing factors. The resulting 2,259 CpG sites were further analysed using principal component analysis (PCA) with the purpose of identifying sites contributing to maximum variance in classifying exposed sites into young and old groups across ethnicities. After testing several thresholds, a PCA loading cut-off of 0.024 was applied to the first component resulting in 310 probes. In addition, 233 probes from an Age group: Site interaction model were also included, as these demonstrated a greater change with age in sun-exposed samples compared to sun-protected samples, thereby reflecting exogenous ageing. This resulted in an exogenous age list comprised of 505 CpG sites.

An additional 156 photodamage-sensitive probes were included in this extrinsic age list. These sites were found to be differentially methylated in young to old sun-exposed sites as well as sun-exposed to sun-protected sites in younger subjects. These sites were also found to change in the expected direction over 5 months in a sun-exposed site and to a lesser extent on arms protected by an SPF product in a previous study (Furtak exposed dataset; E-MTAB-14620) (Furtak et al. (under review)), indicating that these sites are primarily reflecting exogenous ageing driven methylation changes.

Out of the initial 656 probes, 583 were selected after passing quality control checks in the Bormann et al dataset. Subsequently, a ridge regression age prediction model – implemented with the *glmnet* package in R (version 2.0-16) (Friedman et al., 2010) – was employed using these 583 probes and two training datasets: the Furtak exposed dataset (Furtak et al. (under review)) and the Bormann dataset (Bormann et al., 2016).

The age prediction model utilized 4-fold cross-validation and determined each probe's direction of change with age, as reflected in their model coefficients. Ridge regression was chosen as it ensured that all probes retained non-zero coefficients. This approach allowed us to capture and understand the behaviour of each probe comprehensively, as no probe was excluded from the model.

To then determine each probe’s direction of change with photo-exposure, differential methylation analysis between photo-exposed and photo-protected skin samples was assessed in the Furtak exposed and Furtak protected datasets. This was achieved by applying empirical Bayes moderated t-tests to the model coefficients for differential methylation and using an FDR threshold of 5%. Probes from the aforementioned 583-probe set that also showed significant differential methylation (adjusted p-value < 0.05) and shared the same sign between their differential methylation and initial ridge regression model coefficient were exclusively selected, resulting in a list of 308 probes. We then trained 5 elastic net regression models (*glmnet* package in R) on these two training datasets using the 308 probes to further shortlist the most impactful probes.

Only 21 probes were found to have non-zero coefficients across all five models. Consequently, 28 probes with non-zero coefficients in at least two of the five models were selected for further refinement using an elastic net model. This approach was preferred over ridge regression to allow for additional elimination of unnecessary probes. After training three elastic net regression models on these 28 probes, it was observed that all three models agreed on the elimination of four additional probes, although they did not share the exact same coefficients. To produce the final model, the coefficients for the remaining 24 probes and the model intercept were averaged across these three models.

**Generation of the Endogenous Skin Ageing epigenetic clock**

To generate an Endogenous Skin Ageing epigenetic clock, we selected CpG site probes by conducting a differential methylation analysis between young and old samples from sun-protected sites in the Furtak young-old dataset, as described previously (Furtak et al. (under review)). CpG site probes also significantly different between young and old sun-protected and sun-exposed body sites were excluded. This yielded a list of 1,575 CpG sites that better reflected endogenous ageing. Next, PCA analysis was conducted to identify which of these 1,575 CpG sites are contributing to the maximum variance in classifying sun-protected sites into young and old groups. A PCA loading cut-off of 0.030 was applied to the first component resulting in 322 probes.

Next, an elastic net model was utilised on the training datasets to subset the 322 CpG sites into a list of endogenous age predictors. A total of 3 iterations were performed. For each subsequent interaction, the identified predictors from the previous iteration were excluded. The first iteration identified 36 sites, the second identified 53 sites and the third and last iteration identified 25 sites. These 114 probes were then screened against three datasets: the Vandiver dataset (Vandiver et al., 2015); MuTHER dataset (Nica et al., 2011; Roos et al., 2017; Shore et al., 2024); and the Shyne dataset (Shyne et al., 2024), which resulted in the identification of 107 probes that appeared in all datasets.

Prediction models for biological age were then derived based on these 107 probes using a combination of four datasets: Bormann dataset (n=59); the sun-protected subset of the Furtak young old dataset dataset (n=24); MuTHER dataset (n=48); and the Shyne dataset (n=48). These datasets ensured that the probes selected will be trained to predict age from both epidermal and full thickness skin samples across different body sites.

To achieve robust and parsimonious prediction of biological age using a minimal set of CpG probes, we conducted a 2-step variable selection process, which consisted of an initial probe selection followed by coefficient optimization. First, an elastic net regression was employed to shortlist a consistent set of probes, utilizing 4-fold cross validation to optimize the hyperparameters. A total of 3 runs were conducted for this preselection, which identified 41 probes with non-zero model coefficients across all 3 runs. We then further filtered this list down to the most impactful probes by selecting only those on the same order of magnitude as the most impactful probe, (cg15393490, whose coefficient ranged from -66.81 to -68.20 across the 3 runs). After selecting probes whose maximum coefficient absolute value across all 3 runs was greater than 0.1 times the minimum coefficient absolute value of the top probe (>6.68), 25 probes remained. Finally, ridge regression was applied 3 times on these 25 probes to determine the optimal coefficients for the final model. Incidentally, all 3 runs produced the exact same model coefficients, and no further adjustments were required.

**Fibroblast culturing and DNAm profiling**

Human dermal fibroblasts were grown at passage 2 and cultured in an atmosphere of 5% CO2 and atmospheric oxygen in Growth medium (DMEM supplemented with 10% Foetal calf serum and 1% penicillin/streptomycin) until they reached confluence. Each cell strain was sub-cultured to 70% confluence and then growth in maintenance medium (DMEM supplemented with 1% Foetal calf serum) to slow the growth of the cells to more *in-vivo* relevant replication rates. Cells were then harvested after 2 weeks in the slower growing medium, counted and DNA was extracted from the cell pellets using the Qiagen DNeasy Blood & Tissue Mini kit (#69504), according to the manufacturer’s instructions. DNA methylation profiles were generated with the Illumina 450K array using the extracted DNA samples according to manufacturer’s instructions.

**Bibliography**

Ayer, J., Ahmed, A., Duncan‐Parry, E., Beck, P., Griffiths, T. W., Watson, R. E. B., & Griffiths, C. E. M. (2018). A photonumeric scale for the assessment of atrophic facial photodamage. The British Journal of Dermatology, 178(5), 1190–1195. 10.1111/bjd.16331

Bormann, F., Rodríguez-Paredes, M., Hagemann, S., Manchanda, H., Kristof, B., Gutekunst, J., Raddatz, G., Haas, R., Terstegen, L., Wenck, H., Kaderali, L., Winnefeld, M., & Lyko, F. (2016). Reduced DNA methylation patterning and transcriptional connectivity define human skin aging.15(3), 563–571. 10.1111/acel.12470

Friedman, J., Hastie, T., & Tibshirani, R. (2010). Regularization Paths for Generalized Linear Models via Coordinate Descent. J Stat Softw, 33(1), 1–22.

Furtak, A., Shen, J., Ramos del Caño, L., Bappal, A., Dadd, A. W., Lim, F. L., Rocha, S., & Gunn, D. A.Epigenetics changes differentiate between endogenous and photo-ageing in skin and can detect sun-protection factor cream effects in a 5-month intervention study.

Griffiths, C. E. M., Wang, T. S., Hamilton, T. A., Voorhees, J. J., & Ellis, C. N. (1992). A Photonumeric Scale for the Assessment of Cutaneous Photodamage. Archives of Dermatology, 128(3), 347–351. 10.1001/archderm.1992.01680130061006

Horvath, S., Oshima, J., Martin, G. M., Lu, A. T., Quach, A., Cohen, H., Felton, S., Matsuyama, M., Lowe, D., Kabacik, S., Wilson, J. G., Reiner, A. P., Maierhofer, A., Flunkert, J., Aviv, A., Hou, L., Baccarelli, A. A., Li, Y., Stewart, J. D., . . . Raj, K. (2018). Epigenetic clock for skin and blood cells applied to Hutchinson Gilford Progeria Syndrome and ex vivo studies. Aging (Albany NY), 10(7), 1758–1775. 10.18632/aging.101508

Nica, A. C., Parts, L., Glass, D., Nisbet, J., Barrett, A., Sekowska, M., Travers, M., Potter, S., Grundberg, E., Small, K., Hedman, Å K., Bataille, V., Tzenova Bell, J., Surdulescu, G., Dimas, A. S., Ingle, C., Nestle, F. O., di Meglio, P., Min, J. L., . . . Spector, T. D. (2011). The Architecture of Gene Regulatory Variation across Multiple Human Tissues: The MuTHER Study. PLoS Genet, 7(2)10.1371/journal.pgen.1002003

Roos, L., Sandling, J. K., Bell, C. G., Glass, D., Mangino, M., Spector, T. D., Deloukas, P., Bataille, V., & Bell, J. T. (2017). Higher Nevus Count Exhibits a Distinct DNA Methylation Signature in Healthy Human Skin: Implications for Melanoma. The Journal of Investigative Dermatology, 137(4), 910–920. 10.1016/j.jid.2016.11.029

Shore, C. J., Villicaña, S., El-Sayed Moustafa, J. S., Roberts, A. L., Gunn, D. A., Bataille, V., Deloukas, P., Spector, T. D., Small, K. S., & Bell, J. T. (2024). Genetic effects on the skin methylome in healthy older twins. American Journal of Human Genetics, 111(9), 1932–1952. 10.1016/j.ajhg.2024.07.01

Shyne, A., Barnes, B., Gunn, D., Watson, R., Farrar, M., Nair, N., & Orozco, G. (2024). 456 Comparing the DNA methylation and transcriptomic signature of the epidermis and dermis with regards to age and the UV response. The Journal of Investigative Dermatology, 144(12)10.1016/j.jid.2024.10.467

Vandiver, A. R., Irizarry, R. A., Hansen, K. D., Garza, L. A., Runarsson, A., Li, X., Chien, A. L., Wang, T. S., Leung, S. G., Kang, S., & Feinberg, A. P. (2015). Age and sun exposure-related widespread genomic blocks of hypomethylation in nonmalignant skin. Genome Biology, 16(1)10.1186/s13059-015-0644-y
